# PRAME immunohistochemistry demonstrates high diagnostic accuracy for distinguishing melanoma from nevi but no observed association with pT stage in cutaneous melanoma: a Vietnamese clinicopathological study

**DOI:** 10.1186/s13000-026-01806-7

**Published:** 2026-07-15

**Authors:** Giang Huong Tran, Duyen Hoang-My Truong, Lam Ngoc Nguyen, Tu Anh Thai, Khoa Anh Luong

**Affiliations:** 1 Department of Histology, Embryology, and Pathology – School of Medicine, University of Medicine and Pharmacy at Ho Chi Minh City, Ho Chi Minh City, Vietnam; 2 Department of Pathology, University Medical Center Ho Chi Minh City, 215 Hong Bang Street, Cho Lon Ward, Ho Chi Minh City, 700000 Vietnam; 3 Department of Pathology, Oncology Hospital, Ho Chi Minh City, Vietnam

**Keywords:** PRAME immunohistochemistry, Melanoma, Nevi, Sensitivity, Specificity, Tumor stage

## Abstract

**Background:**

PRAME immunohistochemistry is a useful adjunct in distinguishing melanoma from benign melanocytic lesions, but its clinicopathologic associations remain incompletely understood, particularly in underrepresented populations.

**Methods:**

This retrospective study included 90 primary melanomas and 90 melanocytic nevi from a Vietnamese cohort. PRAME expression (clone EPR20330) was assessed using a semi-quantitative scoring system: 0 (0%), 1+ (1–25%), 2+ (26–50%), 3+ (51–75%), and 4+ (> 75% of tumor cells with nuclear positivity), with 4 + defined as high expression. Diagnostic performance was evaluated using receiver operating characteristic (ROC) analysis, and associations with clinicopathologic variables were analyzed using logistic regression.

**Results:**

The mean age of melanoma patients was 56.4 ± 15.9 years, with a median tumor size of 30 mm. Nodular melanoma was the most common subtype (60%), and mucosal melanoma accounted for 15.6%. Adverse features were common, including Breslow thickness > 1 mm (87.8%), mitotic rate > 1/mm² (85.6%), and ulceration (36%). High PRAME expression was observed in 73.3% of melanomas, whereas 97.8% of nevi were negative and none showed PRAME ≥ 2+. PRAME demonstrated excellent diagnostic performance (AUC 0.936), with sensitivity, specificity, and accuracy of 87.8%, 97.8%, and 92.8% at a cutoff of ≥ 1+. Tumor size > 12 mm was independently associated with high PRAME expression in the overall cohort but not in cutaneous melanoma. No statistically significant associations were observed between PRAME expression and tumor stage in cutaneous melanoma.

**Conclusions:**

In this predominantly advanced-stage Vietnamese cohort, PRAME immunohistochemistry showed high sensitivity and specificity in distinguishing melanoma from nevi; its performance in early or thin melanomas and its broader clinicopathologic significance remain to be clarified in future studies.

## Introduction

PRAME (PReferentially expressed Antigen in MElanoma), also known as CT130, MAPE, or OIP-4, belongs to the cancer–testis antigen (CTA) family [1]. It was first identified by Ikeda et al. in 1997 in metastatic melanoma [2]. PRAME is minimally expressed in normal tissues, with restricted expression in immune-privileged organs such as the testis, ovary, placenta, adrenal gland, and endometrium, but is strongly expressed in various malignancies, including melanoma, breast carcinoma, non-small cell lung cancer, head and neck squamous cell carcinoma, renal cell carcinoma, ovarian carcinoma, myxoid liposarcoma, and several leukemias [3]. 

In routine dermatopathology, most melanocytic lesions can be diagnosed based on conventional hematoxylin–eosin (H&E) morphology. However, distinguishing melanoma from benign melanocytic nevi can be diagnostically challenging, particularly in cases with overlapping cytological and architectural features. Traditional melanocytic markers such as S100, Melan-A, and SOX10 exhibit high sensitivity but limited specificity, whereas HMB45, despite higher specificity, has reduced utility in superficial or fragmented specimens [4, 5]. Consequently, there remains a critical need for highly specific immunohistochemical markers to aid in the distinction between melanoma and benign melanocytic lesions.

Recent studies have highlighted PRAME as a promising diagnostic marker, showing diffuse nuclear expression in melanoma but absent or focal staining in nevi [6, 7]. Beyond diagnosis, PRAME expression has also been associated with adverse prognosis and increased metastatic risk in uveal melanoma, where it has been identified as an independent prognostic biomarker, although such findings may not be directly extrapolated to cutaneous or mucosal melanoma [8]. Moreover, PRAME has emerged as a potential immunotherapeutic target, with PRAME-directed vaccines and T-cell receptor–engineered therapies under active investigation [9, 10]. 

To date, no study has systematically evaluated PRAME expression in melanocytic tumors in the Vietnamese population. This study aims to assess the diagnostic performance of PRAME immunohistochemistry and its association with clinicopathologic features of melanoma.

## Materials and methods

### Study population

This retrospective study comprised 90 cases of primary melanoma and 90 cases of nevi, classified according to the WHO Classification of Tumours: Skin Tumours, 5th edition [11]. All included cases had complete clinicopathological data, including age, sex, tumor location, and tumor size.

Cases were excluded if tissue fixation was suboptimal or if the remaining material was insufficient for immunohistochemical evaluation.

The final cohort included 90 primary melanomas, spanning multiple histological subtypes as detailed in the Results section, with no Spitz melanoma or blue nevus-associated melanoma identified. The 90 nevi comprised intradermal nevi (*n* = 45, 50.0%), dysplastic nevi (*n* = 17, 18.9%), blue nevi (*n* = 15, 16.7%), compound nevi (*n* = 8, 8.9%), junctional nevi (*n* = 3, 3.3%), and Spitz nevi (*n* = 2, 2.2%).

### Immunohistochemistry

Formalin-fixed, paraffin-embedded tissue sections (3 μm) were mounted on positively charged glass slides, dried overnight at 37 °C, and incubated at 57 °C for 30 min. Immunohistochemical staining was performed on the fully automated BenchMark ULTRA platform (Roche/Ventana) using the OptiView DAB IHC Detection Kit. A ready-to-use rabbit monoclonal anti-PRAME antibody (clone EPR20330; Roche/Ventana) was applied according to the manufacturer’s validated protocol. Testicular tissue and previously confirmed PRAME-positive melanoma specimens served as positive controls [12, 13], and PRAME expression was identified by nuclear staining. In pigmented cases, the nuclear DAB (diaminobenzidine) signal from PRAME immunostaining was distinguished from endogenous melanin — a brownish-yellow to tan cytoplasmic pigment — by its nuclear localization [14, 15]. 

Automated staining included deparaffinization, heat-induced antigen retrieval with CC1 buffer at 100 °C for 64 min, inhibition of endogenous peroxidase activity, and incubation with the primary antibody for 32 min at 37 °C. Signal detection was achieved using the OptiView system with diaminobenzidine as the chromogen, followed by counterstaining with Hematoxylin II and Bluing reagent. Slides were subsequently dehydrated, cleared, and coverslipped for histopathological evaluation.

### Data collection

Clinical data were retrospectively retrieved from medical records and categorized as binary variables, including age (≤ 30 vs. >30 years), sex (male vs. female), tumor site (extremities vs. non-extremities), and tumor size (≤ 12 vs. >12 mm, based on Ma et al., 2020 [16]). All variables were cross-verified between clinical documentation and pathology reports to ensure data accuracy. Tumor size refers to the gross clinical dimension of the primary tumor as recorded in clinical or operative records, distinct from Breslow thickness — a histopathological measurement obtained from microscopic sections.

Histopathological and immunohistochemical features were independently assessed by three board-certified pathologists, and discrepancies were resolved by consensus. Evaluated parameters included histological subtype (mucosal vs. non-mucosal melanoma), mitotic rate (< 1 vs. ≥1 mitosis/mm²), presence of intratumoral pigmentation (absent vs. present), Breslow thickness (≤ 1 vs. >1 mm), ulceration (absent vs. present), and the degree of tumor-infiltrating lymphocytes (TILs) (absent or focal vs. brisk). Tumor stage (pT) was reassessed according to the 8th edition of the American Joint Committee on Cancer (AJCC) staging system in cases with sufficient clinicopathologic information [17]. 

PRAME immunoreactivity was evaluated according to the criteria proposed by Lezcano et al. and classified as follows: 0 (0%), 1+ (1–25%), 2+ (26–50%), 3+ (51–75%), and 4+ (> 75%) of tumor cells showing nuclear positivity [18]. Cases with PRAME expression scores of 4 + were defined as exhibiting high PRAME expression, whereas scores ≤ 3 + were considered low or negative expression.

### Statistical analysis

Data were entered into Microsoft Excel 2021 and analyzed using Stata version 17.0. The diagnostic performance of PRAME in distinguishing melanoma from nevi was evaluated using sensitivity, specificity, accuracy, and receiver operating characteristic (ROC) curve analysis with estimation of the area under the curve (AUC). Associations between PRAME expression and clinicopathologic variables were assessed using univariate and multivariable logistic regression, with results reported as odds ratios (ORs) and 95% confidence intervals (CIs), treating predictors as appropriate for binary or continuous variables. In cutaneous melanoma, the association between PRAME expression (analyzed as ordinal score, binary, and continuous percentage) and pT (grouped as T1–T4) was further evaluated using ordinal logistic regression (proportional odds model) and Spearman rank correlation. All tests were two-sided, with *p* < 0.05 considered statistically significant.

## Results

### Clinicopathologic characteristics of melanoma

The study cohort comprised 90 patients with primary melanoma, with a mean age of 56.4 ± 15.9 years (range: 20–91). The majority of patients were older than 30 years (93.3%), and 58.9% were female. Tumors most frequently involved the extremities (53.3%), followed by other anatomical sites (46.7%). The majority of melanomas measured > 12 mm in size (86.7%). The median tumor size in our cohort was 30 mm, with an interquartile range of 20–45 mm.

**Fig. 1:**
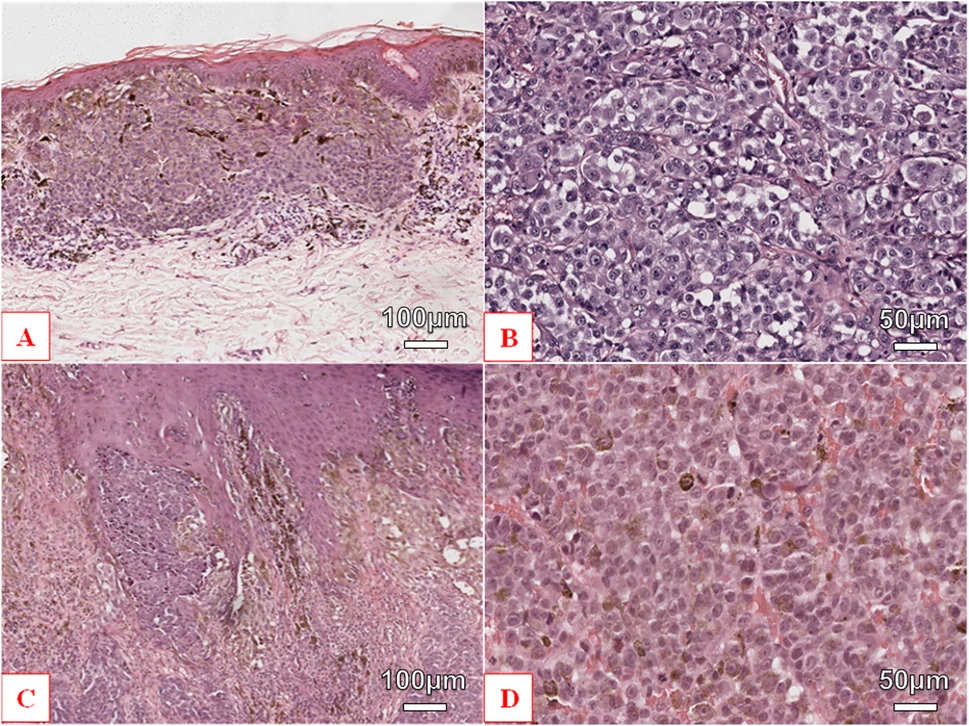
Representative histopathologic images of selected melanoma cases. **A** Superficial spreading melanoma (H&E, ×200); **B** Nodular melanoma (H&E, ×400); **C** Acral lentiginous melanoma (H&E, ×200); **D** Mucosal melanoma (H&E, ×400)

Histologically, nodular melanoma was the most prevalent subtype, accounting for 60% of cases, followed by acral lentiginous melanoma (17.8%). Notably, mucosal melanoma comprised a substantial proportion of the cohort (15.6%), with cases arising from the female genital tract (*n* = 11, 78.6%), including vulvar (*n* = 6), cervical (*n* = 3), vaginal (*n* = 1), and endometrial (*n* = 1) melanomas, as well as from the anorectal region (*n* = 2, 14.3%) and oral cavity (*n* = 1, 7.1%; hard palate). Mitotic activity ≥ 1 mitosis/mm² was observed in 85.6% of tumors, while intratumoral melanin pigmentation was present in 78.9% of cases. Breslow thickness was not assessed in melanoma in situ or mucosal melanoma; among cutaneous melanomas, two cases were unevaluable, and among those with available measurements, 87.8% had a Breslow thickness > 1 mm. Ulceration was identified in 35.6% of cases, and a brisk TILs response was noted in 36.7%. (Table 1) (Fig. 1).

**Table 1:** Summary of clinicopathologic characteristics of melanoma

Characteristics	Summary statistics
Age	56.4 ± 15.9 (20–91)
≤ 30	6/90 (6.7)
> 30	84/90 (93.3)
Female	53/90 (58.9)
Tumor site	
Extremities	48/90 (53.3)
Others	42/90 (46.7)
Tumor size > 12 mm	78/90 (86.7)
Histological types	
Nodular melanoma	54/90 (60)
Acral lentiginous melanoma	16/90 (17.8)
Mucosal melanoma	14/90 (15.6)
Lentigo maligna melanoma	2/90 (2.2)
Superficial spreading melanoma	2/90 (2.2)
Melanoma in situ	2/90 (2.2)
Mitotic Rate ≥ 1 per mm^2^	77/90 (85.6)
Intratumoral pigmentation	71/90 (78.9)
Breslow Thickness > 1 mm	65/74 (87.8)
Ulceration Present	32/90 (35.6)
Tumor-Infiltrating Lymphocytes Present, brisk	33/90 (36.7)

*N* (%) for categorical variables and Mean ± SD (Range) for continuous variables)

### Diagnostic performance of PRAME

**Fig. 2:**
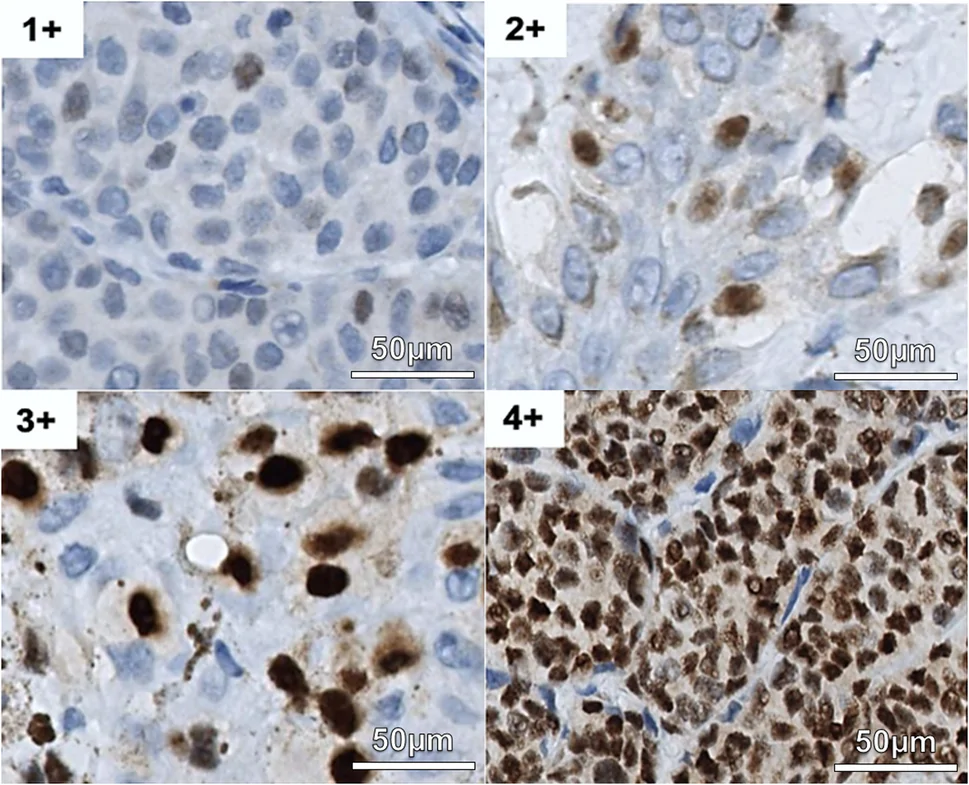
Immunohistochemical images illustrating different PRAME nuclear staining scores (×400)

PRAME staining scores differed markedly between melanoma and nevi. The majority of melanomas exhibited PRAME scores ≥ 3+ (80%), with a predominance of score 4+, whereas nevi were largely negative or demonstrated only minimal staining (score 0–1+), and none showed PRAME expression ≥ 2+. Notably, only two nevi exhibited 1 + PRAME positivity, and both were histologically classified as dysplastic nevi. (Table 2) (Fig. 2).

**Table 2:** PRAME immunohistochemistry: distribution of staining scores and diagnostic performance across different cutoff values

Staining score / Cutoff	Melanoma, *n* (%)	Nevi, *n* (%)	Sensitivity (%)	Specificity (%)	Accuracy (%)
0	11/90 (12.2)	88/90 (97.8)	–	–	–
1+	6/90 (6.7)	2/90 (2.2)	87.8	97.8	92.8
2+	1/90 (1.1)	0/90 (0)	81.1	97.8	89.4
3+	6/90 (6.7)	0/90 (0)	80.0	100	90.0
4+	66/90 (73.3)	0/90 (0)	73.3	100	86.7

**Fig. 3:**
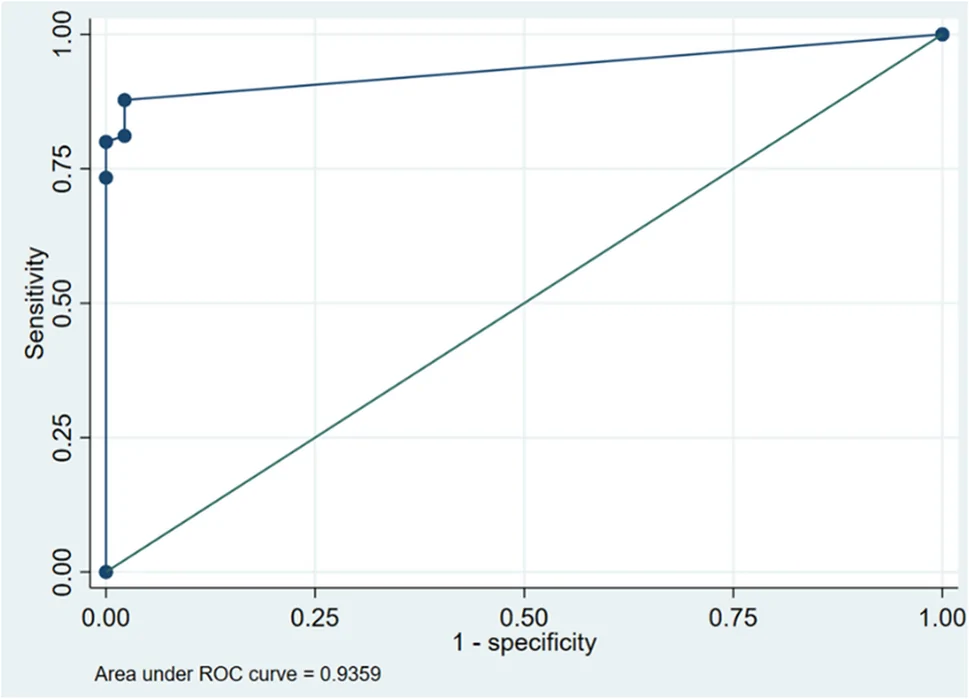
Receiver operating characteristic (ROC) curve of PRAME immunohistochemistry for distinguishing melanoma from nevi

PRAME immunohistochemistry demonstrated excellent diagnostic performance in differentiating melanoma from nevi, with an AUC of 0.936. (Fig. 3) At a cutoff value of ≥ 1+, PRAME achieved the highest overall diagnostic accuracy (92.8%), with a sensitivity of 87.8% and a specificity of 97.8%. Increasing the cutoff value resulted in progressively higher specificity, reaching 100% at cutoffs of ≥ 3 + and ≥ 4+, at the expense of reduced sensitivity. (Table 2)

### Association between PRAME expression and clinicopathologic features

In the overall cohort, univariate analysis identified tumor size > 12 mm as significantly associated with high PRAME expression (OR 5.5, 95% CI 1.5–19.9; *p* = 0.01), whereas no other clinicopathologic variables showed significant associations. Multivariable logistic regression further confirmed tumor size > 12 mm as an independent predictor of high PRAME expression (adjusted OR 5.6, 95% CI 1.3–23.9; *p* = 0.02) (Table 3).

**Table 3:** Univariate and multivariable logistic regression analyses of factors associated with high PRAME expression in melanoma

Variable	Overall cohort (*n* = 90)				Cutaneous melanoma (*n* = 76)			
	Univariate OR (95% CI)	*p*-value	Adjusted OR* (95% CI)	*p*-value	Univariate OR (95% CI)	*p*-value	Adjusted OR* (95% CI)	*p*-value
Age > 30 years	1.7 (0.3–9.4)	0.57	—	—	1.3 (0.2–7.4)	0.81	—	—
Female	2.1 (0.7–5.9)	0.17	1.3 (0.4–4.4)	0.60	2.5 (0.9–7.1)	0.07	2.2 (0.7–6.4)	0.16
Tumor site of extremities	2.1 (0.7–6.0)	0.17	3.1 (0.9–9.9)	0.06	1.7 (0.6–4.6)	0.32	—	—
Tumor size > 12 mm	5.5 (1.5–19.9)	0.01	5.6 (1.3–23.9)	0.02	2.9 (0.7–11.2)	0.12	1.9 (0.4–8.1)	0.41
Mucosal melanoma	3.7 (0.5–30.7)	0.15	7.1 (0.7–73.8)	0.10	—	—	—	—
Mitotic rate > 1/mm²	2.0 (0.5–7.4)	0.32	—	—	2.4 (0.6–8.7)	0.2	1.4 (0.3–5.9)	0.64
Presence of melanin pigment	1.6 (0.5–5.2)	0.45	—	—	1.5 (0.4–5.0)	0.54	—	—
Breslow thickness > 1 mm	2.1 (0.5–8.7)	0.32	—	—	2.1 (0.5–8.7)	0.31	—	—
Ulceration present	1.6 (0.5–4.9)	0.43	—	—	2.3 (0.8–7.3)	0.14	1.9 (0.5–6.5)	0.33
Tumor-infiltrating lymphocytes, brisk	4.4 (0.6–33.4)	0.17	1.1 (0.3–3.9)	0.80	0.5 (0.2–1.4)	0.18	0.9 (0.3–2.8)	0.82
Mitotic count**	1.04 (0.96–1.13)	0.35	—	—	1.01 (0.92–1.10)	0.87	—	—

* Only variables entered into the multivariable model are shown with adjusted ORs

**Mitotic count was additionally analyzed as a continuous variable

However, in the subgroup analysis restricted to cutaneous melanoma (*n* = 76), no clinicopathologic variables were significantly associated with high PRAME expression. Tumor size > 12 mm showed a reduced effect size and was no longer statistically significant (adjusted OR 1.9, 95% CI 0.4–8.1; *p* = 0.41). A separate analysis of mucosal melanoma was not performed due to the small sample size (*n* = 14), among which 12 cases exhibited strong PRAME expression, limiting the ability to conduct meaningful statistical comparisons (Table 3).

With regard to mitotic activity, univariate and multivariable analyses showed that mitotic activity ≥ 1/mm² was not significantly associated with high PRAME expression in either the overall cohort or the cutaneous melanoma subgroup. Similarly, when analyzed as a continuous variable, no significant association with PRAME expression was observed in either group (Table 3).

In the cutaneous melanoma subgroup with complete TNM data (*n* = 74), pT stages were distributed as follows: pT1a (*n* = 4), pT1b (*n* = 5), pT2a (*n* = 1), pT2b (*n* = 2), pT3a (*n* = 11), pT3b (*n* = 4), pT4a (*n* = 26), and pT4b (*n* = 21). For analysis, pT categories were grouped into T1–T4 stages. We further evaluated the association between PRAME expression and tumor stage in cutaneous melanoma, and no significant association was identified across all analytical approaches. In ordinal logistic regression with pT treated as an ordered outcome (T1–T4), PRAME score (0–4+) was not associated with pT stage (OR 0.89, 95% CI 0.64–1.23; *p* = 0.479), with similar results observed using a cutoff of 4+ (OR 1.08, 95% CI 0.39–2.99; *p* = 0.877). When analyzed as a continuous variable, the percentage of PRAME-positive tumor cells was likewise not associated with pT stage (OR per 5% increase 1.005, 95% CI 0.938–1.076; *p* = 0.894), and Spearman correlation analysis demonstrated no monotonic relationship (ρ = 0.067; *p* = 0.568). Additional analyses using alternative dichotomized pT thresholds (e.g., T1 vs. ≥T2 and ≤T2 vs. ≥T3) likewise showed no significant association with PRAME expression.

## Discussion

In the present study, the median tumor size of melanoma was greater than that reported by Ma Q et al. based on the SEER database from 2004 to 2016 (median: 11 mm; interquartile range: 7–20 mm) [16]. In parallel, our cohort demonstrated a predominance of melanomas with a Breslow thickness > 1 mm, a pattern that contrasts with most international series. Specifically, Giavina-Bianchi MH et al. reported that lesions ≤ 1 mm accounted for 49.4% of cases [19], and similarly, Leiter et al. observed a comparable proportion of 52.6% [20]. Furthermore, ulceration was identified in 36% of melanomas in our study, exceeding the rates reported by Lee et al. (29.5%) and Fujisawa et al. (27.9%) [21, 22]. Collectively, these findings indicate that adverse prognostic features were more prevalent in our study population than in previously published international cohorts, strongly suggesting a tendency toward diagnosis at more advanced disease stages, consistent with a high proportion of advanced pT categories (pT3–pT4), which accounted for 83.8% of cases in our cohort.

Several factors may underlie this observation. Our institution functions as a tertiary referral center, where patients with advanced or complex disease are disproportionately represented. Limited public awareness of melanoma, compounded by the anatomically concealed location of certain lesions (e.g., plantar surface), financial constraints, and psychological reluctance to seek medical care, likely contribute to delayed presentation. The absence of structured screening programs and limited availability of dermoscopy further exacerbate diagnostic delays. Additionally, suboptimal biopsy practices and inadequate clinicopathological communication remain challenges in the Vietnamese healthcare setting, potentially compromising timely and accurate diagnosis.

In our study, nodular melanoma was the predominant histological subtype, accounting for 60% of cases. In contrast, a large-scale study of 7,442 Japanese melanoma patients collected between 2005 and 2022 reported acral lentiginous melanoma as the most common subtype (40.8%) [22]. In Western populations, superficial spreading melanoma has frequently been reported as the dominant subtype, as demonstrated in studies by Lezcano et al. (39.3%) and Cazzato et al. (53.5%) [18, 23]. Notably, mucosal melanoma accounted for 15.6% of cases in our cohort, a proportion substantially higher than that reported by Fujisawa et al. (9.5%) and by Anna Carbó-Bagué et al. (1.2%) [22, 24]. These differences are likely attributable to variations in racial and genetic backgrounds, as well as regional differences in patterns of ultraviolet exposure and environmental risk factors. However, the precise contribution of these factors remains incompletely understood and warrants further investigation in future, large-scale, multiethnic studies.

This study provides evidence from Vietnam supporting the diagnostic utility of PRAME immunohistochemistry in distinguishing melanoma from nevi, with high sensitivity and specificity at a cutoff of ≥ 1+. These findings, however, were obtained from a cohort predominantly comprising advanced-stage melanomas, and their generalizability to early or thin melanomas should be interpreted with caution. Although most published reports worldwide have selected a diagnostic cutoff of 4+, as summarized in Table 4, our findings are concordant in demonstrating the strong discriminatory value of PRAME for distinguishing melanoma from benign melanocytic lesions. (Table 4) Importantly, our findings further suggest that even at lower cutoff levels, PRAME may retain substantial diagnostic value.

**Table 4:** Diagnostic value of PRAME in melanoma across selected reports

Report	Year	Cutoff (%)*	Sensitivity (%)	Specificity (%)	Accuracy (%)
Lezcano et al. [18]	2018	> 75	91	100	91
Lezcano et al. [25]	2020	> 75	69	97	90
Raghavan et al. [26]	2020	> 60	92	98	96
Tio et al. [27]	2020	> 75	50	100	67
Lohmann et al. [28]	2021	> 75	67	100	83
Alomari et al. [29]	2021	> 75	53	86	77
Googe et al. [30]	2021	> 75	79	99	91
Harvey et al. [31]	2021	> 75	64	81	76
Gassenmaier et al. [32]	2021	> 75	59	98	74
Fattori et al. [33]	2022	> 75	94	92	93
McBride et al. [34]	2022	> 75	100	100	100
Olds et al. [35]	2022	10 tumor cells/mm	100	98	99
O’Connor et al. [36]	2022	> 75	63	97	87
Santandrea et al. [37]	2022	> 50	87	84	–
Chen et al. [38]	2023	> 75	90	100	–
Bahmad et al. [39]	2023	> 75	90	96	–
Enevoldsen et al. [40]	2024	> 75	70	100	–
Turner et al. [41]	2024	> 75	82	87	–

*Cutoff denotes the percentage of tumor cells showing nuclear PRAME positivity, except as defined by Olds et al. [35]

Beyond sample size considerations, discrepancies among studies may be partly explained by the biological heterogeneity of melanoma. Melanoma represents a highly heterogeneous group of malignant tumors, characterized by marked intertumoral and intratumoral variability in molecular alterations and protein expression [11]. Consequently, reduced or absent PRAME expression in a subset of cases is not unexpected. In addition, several studies have demonstrated that advanced-stage melanoma may undergo phenotypic switching, a process driven by downregulation of microphthalmia-associated transcription factor (MITF), leading to activation of genes associated with invasion and metastasis [42]. Gelmi et al. reported an inverse relationship between MITF and PRAME expression in melanoma: low MITF levels in poorly differentiated tumor cells are associated with increased PRAME expression, whereas high MITF expression correlates with lower PRAME levels [43]. Accordingly, the proportion of advanced-stage tumors included in a given cohort may influence the observed frequency of diffuse PRAME positivity. Furthermore, tumor microenvironmental factors, racial differences, and technical variables—such as antibody selection and immunohistochemical staining protocols—may also contribute to interstudy variability. Taken together, molecular diversity, intratumoral heterogeneity, phenotypic plasticity, and technical factors likely underlie the reported fluctuations in diffuse PRAME positivity across studies. In addition, the lack of uniformity in defining diffuse PRAME positivity may further account for these differences. Future studies with larger cohorts, deeper exploration of the molecular mechanisms underlying PRAME expression, and integration of artificial intelligence–based quantitative assessment of PRAME-positive tumor cells may help establish a standardized and optimal cutoff, thereby enhancing the diagnostic, classificatory, and prognostic utility of PRAME in melanoma.

In the present study, two cases of nevi exhibited low-level PRAME positivity (1+). This finding underscores that, despite its high diagnostic value, PRAME should be interpreted with caution and always within an integrated clinicopathological context. Careful correlation with histopathological features, along with the use of complementary immunohistochemical markers such as HMB45, Ki-67, and p16, is essential, particularly when evaluating diagnostically challenging melanocytic lesions.

In the overall cohort, multivariable logistic regression identified tumor size > 12 mm as the only independent factor significantly associated with high PRAME expression. To date, studies examining the relationship between PRAME expression and tumor size remain limited. Schefler et al. reported that the mean tumor volume of diffusely PRAME-positive melanomas (710.7 mm³) was significantly greater than that of PRAME-negative tumors (510.58 mm³; *p* = 0.011) [44]. In addition, Ma Q et al. demonstrated that a tumor size cutoff of 12 mm had strong prognostic value for melanoma-specific survival, particularly in T1 tumors, where lesions > 12 mm were associated with a markedly increased risk of melanoma-related mortality [16]. Taken together, the association between high PRAME expression and larger tumor size (> 12 mm) observed in the overall cohort may suggest a link with a subset of melanomas exhibiting distinct biological characteristics.

However, whether PRAME expression is directly related to melanoma prognosis remains uncertain and warrants cautious interpretation. In this context, our study did not demonstrate a significant association between PRAME expression and pT stage in cutaneous melanoma. This finding should be interpreted in light of the limited and partly conflicting evidence regarding the prognostic relevance of PRAME in cutaneous melanoma. Ronchi et al., in a study of 69 nevus-associated cutaneous melanomas, reported that PRAME was significantly more expressed in thick invasive melanomas (> T1) than in thin cases (*p* = 0.02) [45]. This apparent discrepancy may reflect differences in case selection, particularly the restriction of their cohort to nevus-associated melanomas, as well as potential biological heterogeneity among melanoma subtypes. Another plausible explanation is the stage distribution of our cohort, in which the majority of cutaneous melanomas were represented by advanced pT categories, potentially limiting the ability to detect stage-related gradients in PRAME expression. Consistent with our findings, Ricci et al. also reported no significant association between PRAME expression and pT stage, albeit in mucosal melanoma of the head and neck region (*p* = 0.184) [14]. Of note, among the three scoring systems evaluated in that study, the cutoff of ≥ 60% proposed by Raghavan et al. yielded higher sensitivity (77.8% vs. 69.4%) and overall accuracy (85.2% vs. 79.6%) than the conventional ≥ 76% threshold of Lezcano et al., with all three systems maintaining 100% specificity, suggesting that optimal PRAME cutoffs may vary according to melanoma subtype and anatomical site [14, 18, 46]. Importantly, the prognostic significance of PRAME remains uncertain. Parra et al. recently reported no significant association between PRAME expression and disease-free survival in cutaneous melanoma [47]. While pT stage and disease-free survival represent distinct clinical endpoints and are not directly comparable, these findings collectively suggest that the relationship between PRAME expression and both direct and indirect prognostic parameters in cutaneous melanoma remains inconsistent and requires further investigation in larger, well-characterized cohorts.

Consistent with this, when the analysis was restricted to cutaneous melanoma, the association between PRAME expression and tumor size was no longer observed. This discrepancy may partly reflect the inclusion of mucosal melanoma in the overall cohort, as the majority of mucosal cases (12/14) in our study exhibited both tumor size > 12 mm and high PRAME expression, thereby potentially influencing the overall results. However, mucosal melanoma itself was not significantly associated with high PRAME expression in either univariate or multivariable analyses, suggesting that the observed association in the overall cohort may be driven by a small subset of cases rather than representing a consistent biological relationship.

Collectively, these findings underscore the impact of tumor subtype heterogeneity on the observed associations. While our results reinforce the strong diagnostic utility of PRAME, they also indicate that its broader clinicopathologic significance remains to be fully clarified.

### Clinical implications

Although PRAME has been investigated in prior studies, its application remains relatively recent in Vietnam, where resource and economic constraints have limited its broader use; thus, our findings provide additional context from a real-world diagnostic setting in an underrepresented population. PRAME immunohistochemistry represents a highly specific and practical adjunct in the diagnostic evaluation of melanocytic lesions, particularly in limited biopsy samples or morphologically ambiguous cases, where diffuse nuclear positivity facilitates confident distinction between melanoma and nevi. Although an association with tumor size was observed in the overall cohort, its clinical significance remains uncertain.

### Limitations and future directions

The retrospective design and relatively limited sample size represent inherent limitations of this study. In addition, the predominance of advanced-stage disease in our cohort may have reduced the ability to detect potential associations between PRAME expression and clinicopathologic variables. An additional technical limitation is the use of single-antigen PRAME immunohistochemistry rather than a double-staining approach combining PRAME with a melanocytic marker such as MART-1 or HMB45. With single-stain PRAME, non-tumoral cells including lymphocytes and fibroblasts may also exhibit PRAME nuclear positivity, potentially confounding the accurate quantification of tumor cell-specific staining and leading to overestimation of PRAME scores — a limitation that can be effectively addressed by adopting a double-stain approach, as previously demonstrated [14, 48–50]. Future multicenter studies with larger cohorts and longitudinal follow-up are needed to better define the clinicopathologic and prognostic relevance of PRAME and its integration with molecular and immune biomarkers. In Vietnam, future efforts should focus on improving melanoma awareness, promoting dermoscopy for early detection, and strengthening collaboration between clinicians and pathologists to enhance diagnostic accuracy and timely management.

## Conclusion

In this Vietnamese cohort comprising predominantly advanced-stage melanomas, PRAME immunohistochemistry demonstrated high sensitivity and specificity in distinguishing melanoma from nevi; whether these findings extend to early or thin melanomas remains an important question for future investigation. The high prevalence of unfavorable clinicopathologic features in our cohort likely reflects delayed diagnosis and highlights ongoing challenges in early melanoma detection. Nonetheless, PRAME associations with clinicopathologic features—including tumor stage—were limited. The observed association with tumor size in the overall cohort was not evident in cutaneous melanoma, and its clinical significance remains uncertain. Further multicenter studies with larger cohorts, standardized scoring, and longitudinal follow-up are needed to better define the clinicopathologic and potential prognostic relevance of PRAME in melanoma.

## Data Availability

No datasets were generated or analysed during the current study.
